# Twenty years of mathematical modeling in ecology: a thematic architecture and macroecological trend analysis

**DOI:** 10.1186/s12862-026-02605-w

**Published:** 2026-10-22

**Authors:** Srinivasarao Thota, Chandan Kumar, P. Shanmugasundaram

**Affiliations:** 1 Department of Mathematics, Amrita School of Engineering, Amrita Vishwa Vidyapeetham, Amaravati, Andhra Pradesh 522503 India; 2 School of Computer Science and Engineering, VIT-AP University, Amaravati, Andhra Pradesh 522237 India; 3 Department of Mathematics, College of Natural & Computational Sciences, MizanTepi University, 21, Tepi Bushira, Tepi, Ethiopia

**Keywords:** Ecological resilience, Climate–ecosystem interactions, Data-driven sustainability, AI-Assisted ecological modeling, Computational ecology, Sustainability analytics

## Abstract

Mathematical modeling in ecology is indispensable for deciphering ecological complexity and guiding sustainable resource governance, yet its explicit alignment with the United Nations Sustainable Development Goals (SDGs) remains insufficiently quantified. Instead of conducting an unrestricted meta-synthesis of the entire field of quantitative biology, this work offers a detailed scientometric and thematic audit of 229 peer-reviewed journal papers indexed in Scopus from 2005 to 2025 (until August 6, 2025) dealing with the explicit formalization of the link between mathematics and ecology. Integrating bibliometric profiling, keyword network co-occurrence, and automated SciVal SDG classification, the analysis delineates three core thematic architectures: biological foundations and experimental calibration, epidemiological and human dimensions, and theoretical mathematical dynamics. Empirical mapping reveals that 116 articles directly align with UN targets, exhibiting heavy concentration in Good Health and Well-being (SDG 3, 40%), Life on Land (SDG 15, 26%), and Decent Work and Economic Growth (SDG 8, 16%). In contrast, there is a continuing presence of target deficiencies at the techno-ecological interface, including in circular bioeconomies, industrial footprints, and clean energy transitions. This study defines methodological strengths, thematic deficits, and a multi-level framework for the implementation of quantitative ecological modeling and transformation to policy mechanisms under the UN 2030 Agenda.

## Introduction

As the world faces increasing worries about environmental degradation and ecological instability, there is a growing need to create sustainable ecologies. Mathematical modeling has become an essential requirement within the realm of ecological studies, facilitating scientists with the tools required to analyze and make predictions regarding biological and environmental interactions in the context of non-linear space and time scales [1]. As ecosystems continue to face threats in terms of climate change, habitat fragmentation, loss of biodiversity, and unsustainable resource use, there has been a need to build robust and predictive ecological models in order to make conservation policies informed and realistic [2]. In ecological mathematical models, computational frameworks have been developed to assist in testing the scenarios regarding the forecast of system states, feedback analysis between multi-trophic levels, and evaluation of conservation scenarios with reduced risks in terms of cost, logistics, and ecology than field experiments [3]. Through the formal mathematical formulation of process dynamics, these architectures provide rigorous mechanisms for biodiversity conservation and adaptive ecological management planning [4].

Mathematical modeling in this context refers to the development and implementation of formal mathematics, which can be defined as deterministic ordinary and partial differential equations, stochastic jump-diffusion systems, cellular automata, individual-based modeling, and network topology algorithms, especially created to model mechanisms of ecological interaction as opposed to purely statistical curve fitting. In the last two decades, mechanistic models have been extensively used in studies of pathogen transmission dynamics, ecotoxicological bioaccumulation, and the feedback loops between the biosphere and atmosphere. Mathematical ecology helps to bridge theory and evidence by applying biological knowledge to local mechanisms that affect global trends.

While there is a fast growth of publications in the interdisciplinary field between mathematics and ecology, the shortage of theme-based systematic reviews which would explore the development, classification, and the future outlook of this interdisciplinary field persists. The lack of such reviews impedes the scientific community from monitoring the emerging interdisciplinary frontiers, identifying bottlenecks in methodologies and comprehensively exploring how quantified modeling leads to sustainability policies on the planet-wide level. The United Nations’ Sustainable Development Goals (SDGs) form an elaborate plan to address environmental, socio-economic and other challenges. While there are a great number of ecological modeling projects that either implicitly or explicitly support the achievement of the SDGs’ objectives by 2030 [5], an empirical overview of how mathematical modeling research can contribute to the UN SDGs has never been developed.

In order to address these gaps and offer an integrative analysis of the field of mathematical modeling in ecology along with the mapping of the empirical SDGs, the current research is formulated through the following three main research questions:

- **RQ1**: What are the macroscopic trends in publications, growth, and citations of mathematical modeling in ecology in the last twenty years?
- **RQ2**: What are the leading research topics and frontier fields of studies in mathematical modeling in ecology?
- **RQ3**: To what degree and on the basis of what thematic mechanisms do papers in mathematical modeling in ecology correlate with the UN SDG framework?

Through the exploration of the following research questions, the study is set to:

1. Conduct a systematic and comprehensive bibliometric review of the peer-reviewed mathematical ecology literature in the last two decades (2005–2025).
2. Analyze the prominent themes, methodological classifications, and emerging research areas in the field.
3. Conduct the quantification and analysis of the distribution of publications on the UN SDGs framework and examine their multi-objective interdependencies.
4. Develop a scalable multi-level approach to dynamic ecological modeling and data assimilation to solve the SDG monitoring gaps in underserved areas.
5. Provide practical insights into translating quantitative ecological models for SDG interventions for the benefit of researchers, funders, and policymakers.

In contrast to an unconstrained meta-synthesis of all quantitative biology, what is presented here is a concentrated and structurally sound audit of the literature that claims itself through the compound terms ‘Mathematical Modeling’ and ‘Ecology’, embodying the very intellectual baseline that connects formal mathematical description with ecology in explicit terms. In this way, thematic science mapping and UN SDG classification are brought together to highlight trends in macroecology research, identify interdisciplinary intersections, and inform future computational sustainability research.

## Literature review

Ecological systems by their very nature are dynamic, non-linear, and complex; therefore, mathematical modeling has historically been the primary scientific tool in understanding these complex systems [6, 7]. The original theories of deterministic logistic growth models and classic predator-prey models served to provide some of the earliest quantification of population dynamics and interspecific relationships [6, 7]. Following from these classic deterministic theories are now stochastic jump-diffusion models, spatially explicit cellular automata models, and structural network models which can elucidate heterogeneity of the landscape, dispersal mechanisms, and ecosystem stability under local disturbances [8]. As humans alter the environment on a continually increasing scale, ecological modeling has evolved to address conservation, ecosystem service conflicts, and ecological risks [2, 3, 5].

Recent studies have shown that the field of mathematical ecology is becoming more interdisciplinary involving disease ecology, climate-ecosystem interaction, microbiology, and aquatic conservation, among others. For example, numerical simulation models have been extensively used for the study of cryospheric processes and river-ice dynamics in the context of global warming [9], competitive dynamics and assembly of communities in soil microbiomes [10], and multiple trophic predator-parasite interactions influencing evolutionary characteristics [11]. Simultaneously, mathematical epidemiology, using compartmental models (e.g., SIR, SEIR), heterogeneous contact networks, and super-spreader effects, is developing into an important translation tool between ecological theory and public health surveillance [12]. Altogether, these developments prove the importance of mathematical models in predicting non-linear ecological regime shifts [1, 5].

Current sustainability efforts have found that their policies should be aligned with the Sustainable Development Goals set forth by the United Nations. In this light, mathematical ecology is key to formulating these policies into tangible and applicable measures. For example, epidemiological modeling and zoonotic spillover surveillance directly underpin Good Health and Well-being (SDG 3) [1, 12]; hydrodynamic and pollutant fate models safeguard freshwater integrity under Clean Water and Sanitation (SDG 6) [9]; dynamic climate–biosphere feedback models inform Climate Action (SDG 13) [2, 3, 9]; marine food-web and pathogen simulations support Life Below Water (SDG 14) [13]; and population viability analyses under habitat fragmentation inform Life on Land (SDG 15) [11, 14]. Nevertheless, most ecological modeling contributions address these targets only implicitly, leaving the literature fragmented and disconnected from multilateral sustainability monitoring mechanisms.

A limited number of scientometric and systematic literature reviews (SLRs) have examined isolated sub-domains of ecological modeling. These existing syntheses predominantly concentrate on disease vector dynamics (e.g., dengue transmission and control) [15], spatial pattern formation mechanisms in vegetation [16], general cross-disciplinary modeling in economics and environment [17], or specialized ecotoxicological models such as microplastic dispersal [18]. However, no prior study has quantitatively delineated the macro-level intellectual architecture of mathematical modeling in ecology, nor evaluated how its core thematic structures map empirically to the UN SDGs. Existing reviews provide fragmented perspectives, omitting how mathematical formulation, biological data integration, and policy-relevant sustainability outcomes coalesce across the discipline. This structural knowledge gap restricts the translational visibility of mathematical ecology in global environmental governance.

Thematic science mapping using keyword co-occurrence topologies and density–centrality quadrant networks provides an established, robust approach for uncovering intellectual trajectories and theme evolution across complex domains [19–21]. Simultaneously, automated SDG mapping algorithms, including machine-learning classifiers developed by Elsevier and Science-Metrix, enable standardized evaluations of scholarly contributions to global sustainability priorities [22–24]. To date, these two analytical viewpoints—the internal intellectual organization of research and its external contribution to societal challenges—have remained entirely disconnected in the mathematical ecology literature.

### Research gap and novelty of this study

A systematic comparison between existing review literature and the analytical scope of the present study reveals four foundational research gaps, summarized in Table 1.

**Table 1:** Research gaps identified in existing literature

Research Dimension	Earlier Evidence	Identified Gap
Mathematical modeling in ecology reviews	Traditional SLRs exist focusing narrowly on specialized sub-domains (e.g., dengue vector control, microplastics) [15, 17].	Lack of an overarching, structured thematic synthesis of the broader mathematical ecology domain.
Bibliometric science mapping	Science mapping has been applied in general sustainability and macro-environmental studies [16].	Absence of two-dimensional centrality–density thematic mapping dedicated to mathematical ecology.
SDG impact assessments	Available in broad sectoral sustainability assessments and regional programming [25].	Complete absence of empirical UN SDG target mapping for mathematical modeling contributions.
Combined thematic + SDG analysis	No integrated studies linking internal thematic architecture with external SDG classifications.	Major knowledge gap: lack of an empirical bridge connecting mathematical typologies to policy targets.

To explicitly resolve each of the four identified gaps in Table 1, this study provides the first integrated scientometric, thematic, and SDG-mapping review of mathematical modeling in ecology. The specific novel contributions of this work are fourfold:

- **Systematic Scientometric Architecture:** Delivers a twenty-year longitudinal audit of publication trajectories, citation aging metrics (MCPY and ACPD), and structural modeling typologies.
- **Thematic Science Mapping:** Uncovers the domain’s intellectual structure via keyword co-occurrence networks and density–centrality quadrant trajectories, resolving emerging frontiers from regressing themes.
- **Empirical UN SDG Alignment:** Establishes the first multi-goal classification and co-occurrence interlinkage network of mathematical ecology across the 2030 Agenda.
- **Operational Translational Framework:** Formulates an actionable, multi-tiered pipeline bridging mechanistic models with real-time empirical data assimilation and regional policy implementation.

## Methodology

**Fig. 1:**
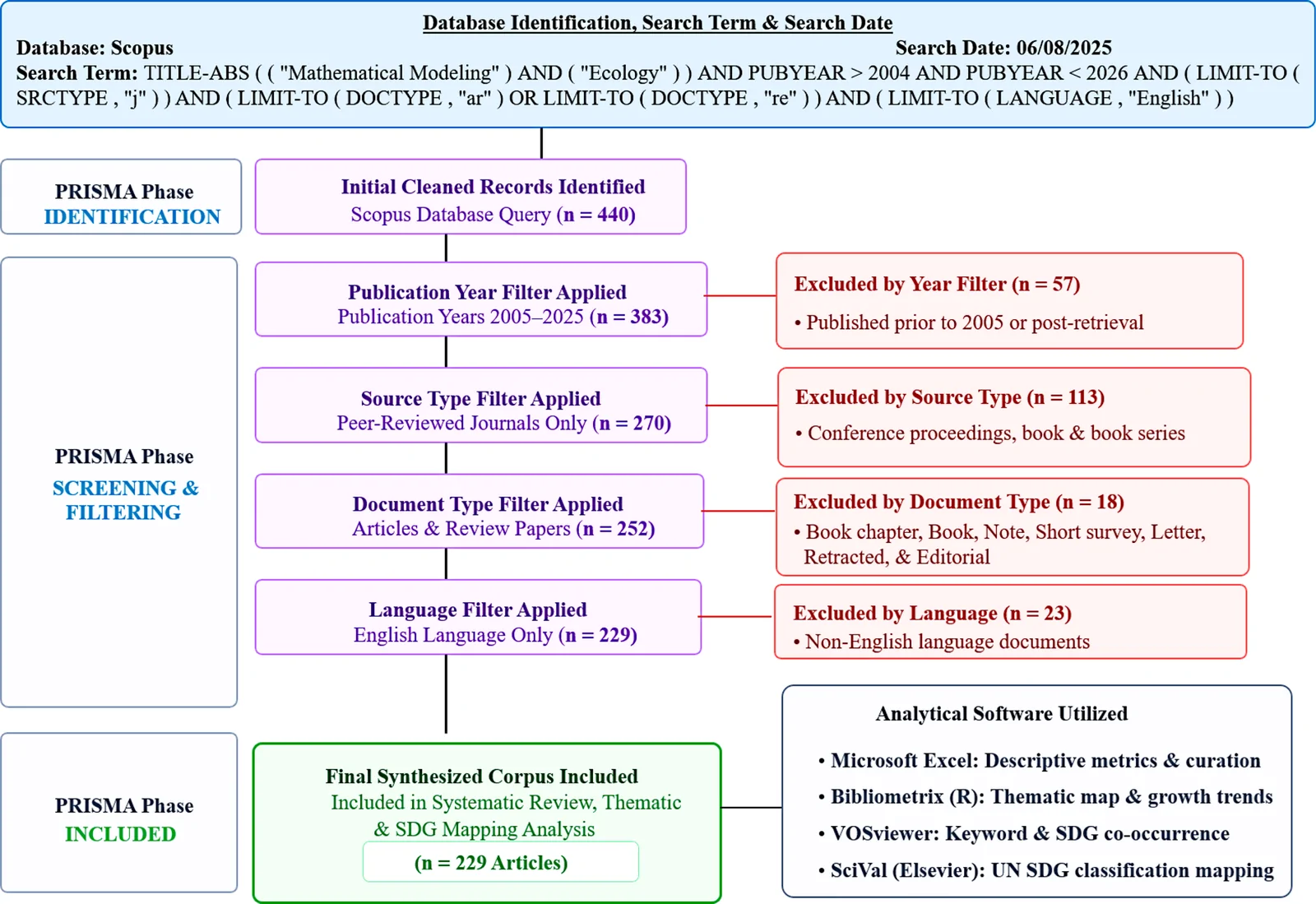
Research design following PRISMA 2020 guidelines

A systematic methodology was established to address the research questions and resolve the empirical gaps identified in Table 1. Figure 1 illustrates the multi-stage research design developed in accordance with the PRISMA 2020 guidelines [26].

### Data collection and preprocessing

A search in the primary literature was carried out on 6th August, 2025. As per the scientometric guidelines [27], bibliographic details were extracted from the Scopus database to perform systematic science mapping studies [28].

It is important to note that the initial syntax search made use of exact phrase matching for TITLE-ABS(”Mathematical Modeling” AND “Ecology”). A comparison of sensitivity was performed by employing a wider version of Boolean search that included related keywords such as “Mathematical Modeling,” “computational,” and “theoretical” in addition to “Ecology” that resulted in a total of 13,642 articles from various fields (Environmental Science, Agricultural and Biological Sciences, Social Sciences, Engineering, Computer Science, and Mathematics). In order to maintain the integrity of the theme and confine the search only to the formalized relationship between mathematical modeling and ecological dynamics, the final 229-article database was kept as the main database to consider. The methodological trade-offs and structural boundaries inherent to this focused query are detailed under the Study Limitations.

The temporal framework covers the period between 2005 and August 6, 2025 (the date of data collection). Due to more than seven months (* > 58%*) of the year 2025 having passed, papers indexed until this date were specifically included to take into account the latest additions and trends, as well as new SDG mapping results (e.g. [29, 30]). Specifically, the relevant publications of 2025 provided essential information on how to define the current research frontier within domains such as hybrid molecular signaling and contemporary scaling theory. It is worth stressing that the number of publications and citations per year of 2025 is the result of partial-year activity only and does not correspond to a complete annual cycle; thus, the count of 2025 publications is partial.

### Exclusion and inclusion

In order to identify only the good and relevant publications, the exclusion and inclusion criteria were used. After using exclusion and inclusion criteria, the dataset has been filtered to 229 articles. The last search term for this research is mentioned below:

**Figure:**
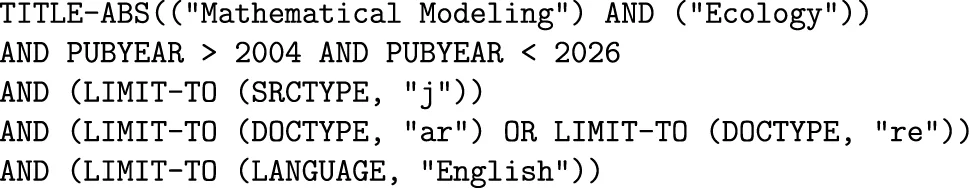

Exclusion criteria included:

- Records published outside the specified publication period (before 2005 or after the retrieval period).
- Conference proceedings, book and book series records, and other non-journal source types.
- Book chapters, books, notes, short surveys, letters, retracted documents, and editorials.
- Non-English language publications.

The inclusion criteria were:

- Articles or review papers published in peer-reviewed journals.
- Publications explicitly addressing mathematical modeling in ecological contexts.
- English-language publications from 2005 to 2025.

To make sure that there is clarity and replicability throughout the screening process, candidate documents were filtered sequentially based on their publication year, source type, document type, and language. In total, the initial Scopus search yielded 440 documents. Out of them, 57 documents were removed due to falling out of the defined publication range. This left 383 documents in total. Exclusion of sources based on their types left us with 270 documents. Further exclusion based on the document type reduced the number to 252 documents, and excluding non-English documents further decreased the number to 229 documents, as detailed in Fig. 1.

**Justification for Database Selection:** The bibliographic search was performed through Elsevier Scopus because of its comprehensive indexation within the realms of applied mathematics and life sciences, as well as its integrated algorithmic connection to Science-Metrix and SciVal SDG taxonomy. While other bibliographic databases like Web of Science Core Collection or PubMed contain equally useful cross-disciplinary coverage, Scopus was chosen as the tool which offered the required metadata and reproducible SDG-mapping infrastructure [31]. This dependence on one bibliographic database is recognized as a boundary condition in the Study Limitations.

**Justification for Document-Type Selection:** Bibliometric and systematic review studies commonly restrict their analyses to peer-reviewed journal articles and review papers to ensure a consistent and reliable scholarly evidence base [32, 33]. Different publication types, including conference proceedings, books, book chapters, editorials, notes, and other document categories, may follow different peer-review procedures, publication practices, metadata structures, and citation patterns. Including such heterogeneous document types may therefore introduce variability in the quality and comparability of the records and affect subsequent citation, thematic, and science-mapping analyses. Accordingly, the present study was restricted to peer-reviewed journal articles and review papers to maintain methodological consistency and improve the reliability and comparability of the bibliometric analysis. The potential exclusion of relevant findings published exclusively in other document types is acknowledged as a boundary condition in the limitations section.

### Evaluation and reporting

The assessment of data and trend analysis is conducted using Microsoft Excel, RStudio [19], VOSviewer [34], and SciVal [35]. Evaluation and reporting are important for interpreting the findings of the study and outlining the limitations.

#### Research publications analysis

Scientometric analysis identifies publication patterns, top journals, authors, institutions, countries, and citation impact [36]. This quantitative layer highlights the trends in research productivity and impact in mathematical ecology. Microsoft Excel and the Bibliometrix R-package are mainly used for this analysis.

**Division of Computational Roles:** To prevent methodological confounding from differing proprietary clustering algorithms, each software package was assigned a discrete operational task:

1. **Bibliometrix (R-package):** Used exclusively for descriptive bibliometric indicators, descriptive tables (Table 2), compound growth rates, and two-dimensional quadrant centrality/density thematic mapping (Fig. 4).
2. **VOSviewer:** Reserved strictly for distance-based spatial visualization of the keyword co-occurrence network (Fig. 3) and the SDG co-occurrence network (Fig. 6) using modularity-based partitioning.
3. **SciVal (Elsevier):** Utilized exclusively for automated retrieval and validation of pre-defined UN SDG categorical classifications.
4. **Microsoft Excel:** Utilized solely for intermediate data curation, sanity checking, and primary charting.

**Table 2:** Descriptive statistics of the 229-article dataset (2005–2025)

Description	Results
Timespan	2005–2025
Sources (Journals, Books, etc.)	177
Documents	229
Annual Growth Rate (%)	0.92
Document Average Age	7.67
Average citations per document	27.13
References	12781
Author’s Keywords (DE)	867
Authors	866
Authors of single-authored documents	32
Single-authored documents	32
Co-Authors per Document	3.9
International co-authorships (%)	33.19
Article	173
Review	56

#### Thematic modeling and mapping

Thematic modeling and mapping are very important in this type of research work because they provide a structured, transparent, and rigorous way to draw connections across diverse research works. The VOSviewer software has been used to identify the core themes of mathematical modeling in ecology and their interconnections. The Bibliometrix R-package tool has been used to generate the thematic mapping to visualise theme evolution across quadrants: motor, basic, emerging/declining, and niche themes. This analysis highlighted the intellectual structure of mathematical modeling in ecology and its dynamic progression.

#### SDG mapping

SDG mapping is important to highlight the contribution of a particular area of research work to the United Nations Sustainable Development Goals (SDGs) and supports policymakers and researchers in making decisions. The SciVal and VOSviewer tools are used to generate the SDG mapping. The three SDG clusters are identified:

- Food Security, Economic Growth, and Climate Action (SDG 2, SDG 8, SDG 13)
- Health, Water, and Ecosystem Sustainability (SDG 3, SDG 6, SDG 14, SDG 15)
- Sustainable Energy, Innovation, and Responsible Consumption (SDG 7, SDG 9, SDG 12)

This mapping shows how mathematical modeling in ecology contributes to different SDG goals.

The three overarching SDG groupings were derived empirically using the VOSviewer modularity optimization algorithm applied to the co-occurrence matrix of mapped SDGs, and conceptually corroborated by the biophysical-socioeconomic stratification of the Stockholm Resilience Centre SDG framework:

1. **Food Security, Economic Growth, and Climate Action (SDG 2, 8, 13):** Reflects resource extraction, economic provisioning, and atmospheric feedbacks.
2. **Health, Water, and Ecosystem Sustainability (SDG 3, 6, 14, 15):** Aligns with the core biophysical biosphere foundation, connecting human and animal health to water and land integrity.
3. **Sustainable Energy, Innovation, and Responsible Consumption (SDG 7, 9, 12):** Represents the technological, computational, and circular industrial interface of ecological modeling.

### Data reliability and validation

To ensure reliability, the bibliometric results were cross-checked manually, and three different domain experts also helped during the cross-checking process. Thematic and SDG mappings were also validated through iterative coding and expert judgment to ensure the clusters genuinely reflect the existing literature.

Validation was conducted by a panel of three independent academic experts: an associate professor in biomathematics, a senior researcher in quantitative ecology, and a specialist in sustainability science metrics. The experts evaluated a stratified random sample of 50 publications (representing *∼*22% of the dataset) across three thematic axes: (i) appropriateness of mathematical formulation, (ii) ecological relevance, and (iii) alignment with assigned SciVal SDG targets. The inter-rater reliability was measured using Fleiss’ kappa (*κ*). The overall measure of reliability obtained is *κ =0.84* (95% confidence interval of [0.76, 0.92]), showing strong agreement. The inconsistencies

## Result and discussion

### Research performance

Table 2 shows the synthesis of the quantitative research performance in order to answer RQ1. This includes the number of publications, publication types, growth trends per year, citation metrics, authorship patterns, and international collaboration dynamics. Descriptive curation and initial metric aggregation were carried out using Microsoft Excel, while the analysis of bibliometric metrics was done using the Bibliometrix R package. The integration of Excel and Bibliometrix ensured the acquisition of the quantitative information about the longitudinal publication trends, citation trends, top journals, countries, and institutions in the field of mathematical ecology.

This set of data demonstrates the compound growth rate of 0.92% per year during the years 2005–2024. Unlike the fast exponential growth (over 5%-8% each year) characteristic for such domains as artificial intelligence and molecular biotechnology, mathematical modeling of ecology is a well-established field associated with constant productivity but not with unlimited growth. It should be noted that the noticeable peaks in 2020 *(n=19),* 2021 (*n=21*) and 2022 (*n=27*) can be attributed to the fast growth in epidemiological-ecological modeling in the course of the pandemic.

To evaluate temporal scientific impact, we distinguish between two standardized metrics:

1. **Mean Citations per Document per Year (MCPY):** Calculated for each publication year *y* as $MCPY_y = \frac{C_y}{N_y \times (T-y+1)}$, where $C_y$ is the cumulative citations accrued by papers published in year *y*, $N_y$ is the number of papers in year *y*, and *(T-y+1)* is the citable age of the publications (with *T=2024*).
2. **Average Citations per Document (ACPD):** Defined globally as total cumulative citations across all records divided by total publications: $\mathrm{ACPD} = \frac{\sum C}{\sum N} = \frac{6213}{229} = 27.13$.

The peak MCPY of 6.81 occurred in the 2008 cohort (largely anchored by Codling et al. [37]), whereas articles published between 2020 and 2023 maintain an average *MCPY ≈ 4.12*, indicating rapid immediate uptake.

**Fig. 2:**
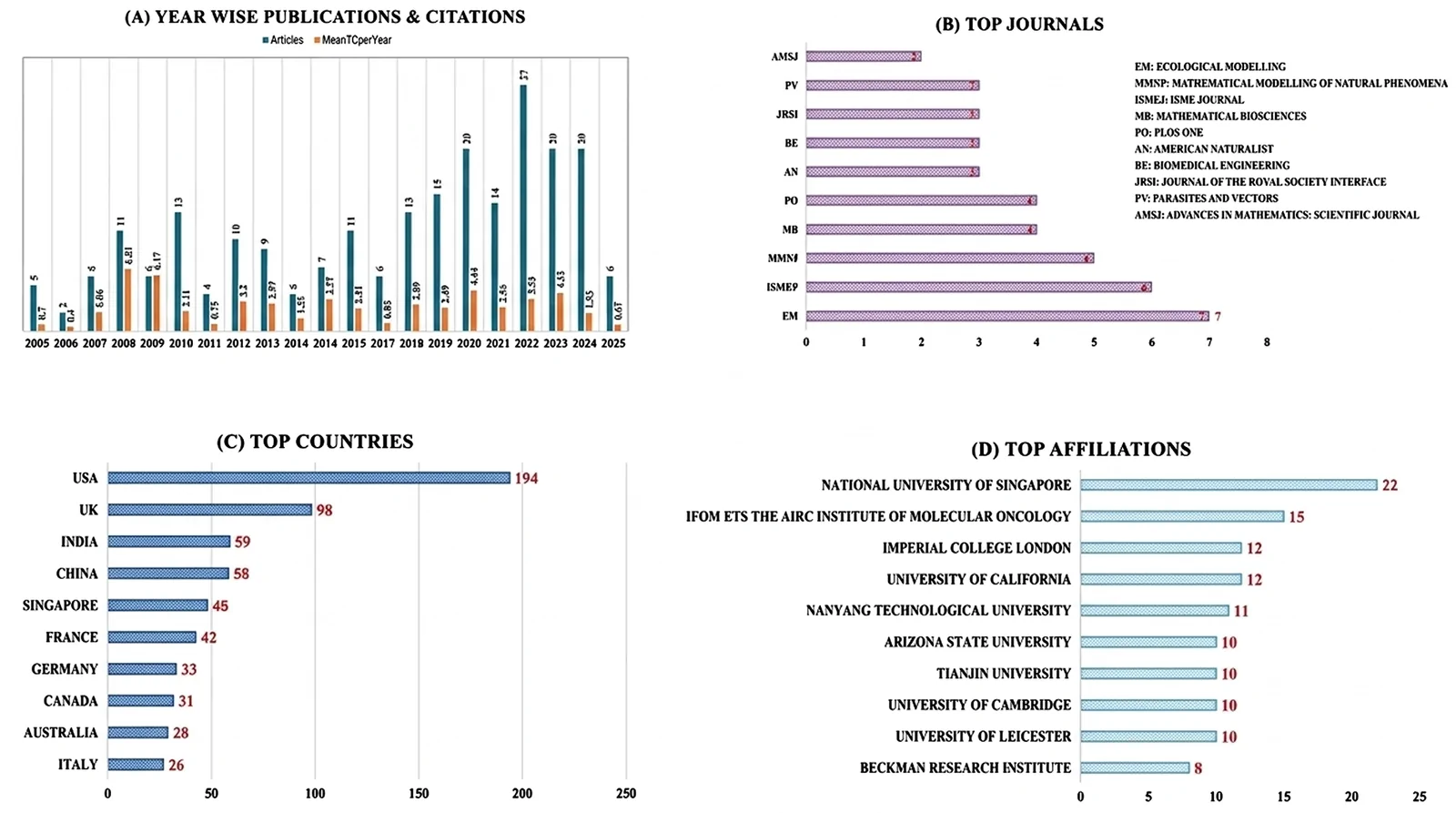
Research performance: (**A**) Annual publications and citations, (**B**) Top 10 publishing journals, (**C**) Top 10 productive countries, and (**D**) Top 10 institutional affiliations

Longitudinal publication and citation trends are illustrated in Fig. 2(A). Publication statistics illustrate how the scientific attention to the field has developed during the last two decades through baselines versus transition periods. As illustrated in Fig. 2(A), the annual number of publications increased to 27 in 2022, then 20 publications each in 2023 and 2024. Instead of showing unstable fluctuations, this indicates that the field has a consistent and developed baseline after research increases during the pandemic period. The maximum mean citations per year was obtained in 2008 (6.81 MCPY), while 2020–2023 cohorts have a stable average close to 4.0 MCPY.

Finding the most productive and credible journals helps to understand where the influential research papers are published. The most influential 10 journals have been presented in Fig. 2(B). These include *Ecological Modelling* with the following two titles *Mathematical Modelling of Natural Phenomena* and *The ISME Journal*. Some of the significant spatial and institutional distributions of the research performance have been presented in Fig. 2(C-D). In terms of geographic distribution of the research performance, the leading country in total number of publications is the United States.

Quantitative metrics provide further structural insights beyond descriptive aggregates. On average, there are 27.13 citations per paper in the data, indicating continuous use in academic literature. Average author *h*-index among the papers under consideration is equal to 25, while the leading single author is E. A. Codling, whose *h*-index is 45. International collaboration network is also strong since 33.19% of all papers include authors from different countries, especially North America and Europe. The count in 2025 (*n=4*) is strictly limited by the mid-year cut-off date (August 6, 2025) in Fig. 2(A) rather than domain contraction; thus, the annualized output is consistent with the baseline.

**Distribution of Modeling Typologies:** From the structural analysis of 229 articles, it is clear that 48.5% of the literature uses deterministic differential equations (ODEs/PDEs), 23.1% uses stochastic and/or probabilistic approach, 17.5% uses agent-based or spatial simulation techniques, and 10.9%.

#### Top cited documents

Citation count is a commonly used proxy measure of academic impact and methodology anchorage [36]. Table 3 illustrates the 10 most cited documents in the corpus. The two most cited papers are Codling et al. (2008) [37] (1,094 citations; 60.78 citations per year) and Lloyd-Smith et al. (2009) [12] (525 citations; 30.88 citations per year). The high citation numbers of these two papers illustrate the importance of movement ecology and epidemic transmission modeling. Papers that have been published for a long time generally have higher cumulative citation counts compared to those that were recently published.

**Table 3:** Top 10 cited documents

TC	Authors Name & Year	TC/Year
1094	Codling et al. (2008) [37]	60.78
525	Lloyd-Smith et al. (2009) [12]	30.88
219	Mukherjee et al. (2015) [38]	19.91
176	Shen, H. T. (2010) [9]	11.00
159	Wu et al. (2020) [39]	26.50
123	Vrancken et al. (2019) [40]	17.57
122	Svensson et al. (2012) [11]	8.71
107	Lyons et al. (2010) [13]	6.69
107	Liu et al. (2022) [41]	26.75
99	Opatowski et al. (2018) [42]	12.38

These top-cited works cover different ecological theories. For instance, Codling et al. (2008) provided a systematic description of random walks in relation to a theoretical framework for animal movement ecology [37]. Lloyd-Smith et al. (2009) contributed the dynamics of superspreading and heterogeneous transmission at the human-animal interface, which became a significant subject during the epidemics [12]. Mukherjee et al. (2015) offered crucial descriptions for environmental modeling and prevention of leachate formation in landfills [38]. Shen (2010) developed numerical algorithms for cold-region rivers ice flow and cryospheric interactions [9]. Lu et al. (2025) offered developments for hybrid signaling and genomics in the field of ecology and cell biology [29]. Other significant studies include Vrancken et al. (2019), dealing with dynamics of synthetic gut microbiome [40]; Svensson et al. (2012) with respect to empirical validation of disturbance-diversity predator-prey models [11]; Lyons et al. (2010) about microscopic island biogeography of aquatic pathogens [13]; Liu et al. (2022) concerning theoretical network resilience [41]; and Opatowski et al. (2018) about pathogen competition dynamics in public health [42]. Taken together, these studies demonstrate the connection between fundamental mathematical theorems, biology experiments, and public health management.

**Underlying Drivers of the Academic Landscape:** The institutional and geographical clustering shown in Figure 2 is due to fundamental institutional drivers behind ecological research funding. The dominance of the USA, UK, and India, in addition to prestigious computing institutions such as the National University of Singapore, Cambridge and Imperial College London, is fueled by continual financial investment into high-performance computing (HPC) clusters and quantitative biology chairs. In addition, major journal outlets, including *Ecological Modelling* and *Mathematical Modelling of Natural Phenomena*, serve as conduits for linking pure mathematical theory with ecological conservation practice. The longevity of the citation life of basic theorems (such as that by Codling et al. [37] on random walks and Lloyd-Smith et al. [12] on superspreading), indicates that current ecological modeling continues to be fundamentally rooted in core theories of movement and transmission, despite a shift towards new threats such as range expansion and global zoonotic monitoring.

### Thematic modeling

In comparison to other generic architectural models of ecology—those which are usually characterized by domination of field empirical botany, community macroecology, and phylogenetic evolution—the architectural divergence of the themes found here can be identified. In particular, mathematical ecology represents a translational framework where compartmental dynamics of epidemiology (Cluster 2) becomes equally important as biological dynamics (Cluster 1) and computational dynamics (Cluster 3). It shows that mathematics applied to ecology is an interdisciplinary effort motivated by practical problems of human–ecosystem–pathogen interactions.

**Fig. 3:**
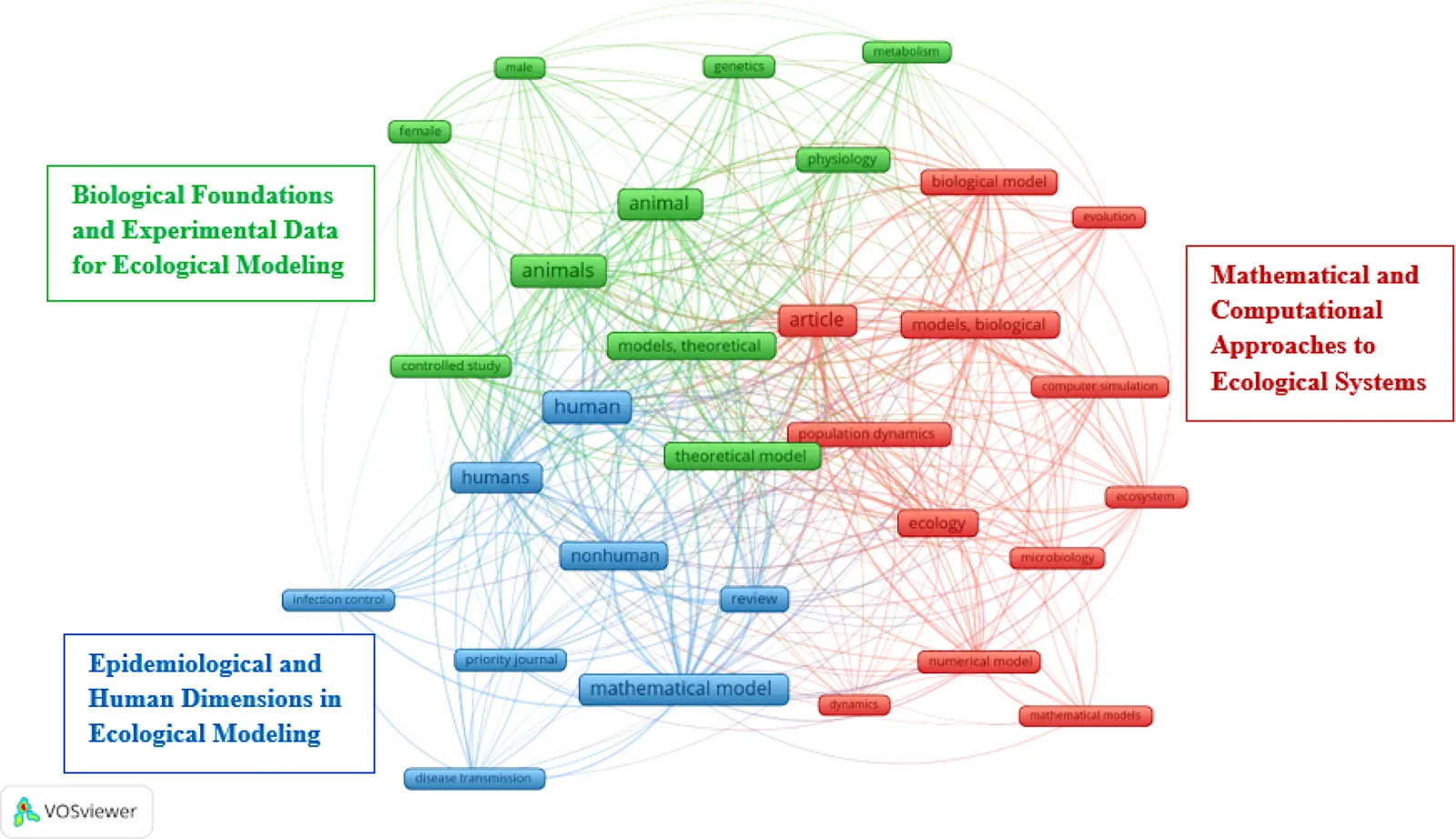
Keyword co-occurrence network with three thematic clusters (VOSviewer)

Thematic analysis uses repetition in the identification of important themes through the recognition and classification of elements of information contained in bibliometric data [20]. By grouping research articles with similarities according to their keyword co-occurrence patterns, thematic analysis is able to identify conceptual themes, trace the historical development of the field, and locate gaps in existing knowledge [20]. VOSviewer was used to construct the keyword co-occurrence network [21]. Three clusters were found: (1) Biological Foundations (Green), (2) Epidemiology and Human Aspect (Blue), and (3) Theoretical and Mathematical Ecology (Red) as illustrated in Fig. 3.

For generating the co-occurrence network for the keywords using VOSviewer (version 1.6.20), the co-occurrence technique was employed on the basis of keywords retrieved from the references. The threshold value of occurrences was defined to eliminate peripheral nodes that were occurring rarely or had some unique features, leaving behind only a stable core network of high-frequency nodes which constitute the basic structure of the field. Link weight between the nodes was calculated by applying association strength normalization, and modularity-based graph clustering was employed.

The three clusters demonstrate the interplay between biology, epidemiology and mathematical modeling:

- **Cluster 1 (Green)—Biological Foundations of Ecological Modeling:** This cluster pays particular attention to keywords like *animals, male, female, genetics, physiology,* and *metabolism*. It is dedicated to experimental biological studies which produce necessary parameterized information for ecological models. Growth rates, reproduction and genetic diversity experiments are performed to provide the needed ecological variables to make ecological models valid and biologically reasonable.
- **Cluster 2 (Blue)—Epidemiological and Human Aspects:** Characterized by keywords like *disease transmission, infection control, humans,* and *non-human*, this cluster stresses the close link between population ecology and epidemiology. Human-centered models, zoonotic spillover phenomena and compartmental system (SIR, SEIR) are some typical features of this cluster.
- **Cluster 3 (Red)—Theoretical and Mathematical Ecology:** Keywords such as *ecology, population dynamics, numerical models,* and *computer simulation* reveal the key computational background of ecological sciences. Deterministic and stochastic approaches are covered in this cluster.

Together, these clusters illustrate that ecological modeling is, by definition, an interdisciplinary field that combines biology, epidemiology, and mathematics to create predictive models.

#### Thematic cluster analysis

In this section is described the deep analysis of the themes that appeared based on the co-occurrence of keywords and bibliometric map. In each of the clusters presented below, we observe a unique field that connects theory of mathematics, biological facts, and sustainable applications. Seminal works and cutting-edge research (2020–2025) were considered for each of the clusters below.

#### Biological foundations of ecological modeling

The Biological Foundation of Ecological Modeling theme integrates biological, metabolic, and genetic information into mathematical equations to ensure that ecological models are biologically rooted. The highly-cited works of Mukherjee et al. [38], Shen [9], and Vrancken et al. [40] have played crucial roles in structuring this theme. For example, Mukherjee et al. [38] formulated equations related to the transport of contaminants in the environment and leachates from landfills, offering fundamental models in pollution risk assessment. Also, Shen [9] has formulated state-of-the-art numerical models of cryospheric and cold-region river-ice processes, illustrating the significance of physics-based models in climate feedback analysis. Moreover, Vrancken et al. [40] contributed to synthetic microbial ecology using dynamic mathematical models and microbial and virome experiments.

The recent modeling approaches have moved to molecular and cellular levels. Lu et al. [29] developed hybrid mathematical models of molecular signaling pathways within ecology, demonstrating a tendency towards convergence of the frameworks of ecological and biomedical modeling. The author of the resource-competition equations, Tilman [10], is the one whose equations are developed by modern authors in order to describe the interaction of bacteria and fungi as well as the equilibrium of soil microorganisms. Thus, these developments can be regarded as an evolution from macro-level empirical modeling towards micro-level individual modeling.

#### Epidemiological and human dimensions in ecological modeling

The cluster on Epidemiological and Human Dimensions emphasizes the use of ecological modeling tools directly in the context of infectious diseases and global public health. The three seminal articles that can be identified in the thematic area include Lloyd-Smith et al. (2009) [12], Opatowski et al. (2018) [42], and Wu et al. (2020) [39], and they all represent the application of ecological principles to the transmission dynamics of pathogens within host populations. In their study, Lloyd-Smith et al. [12] developed a theory of superspreading events and modeled individual transmission heterogeneity in a human-animal interface with stochastic compartmental models. Opatowski et al. [42] used the idea of ecological frameworks for multiple pathogens to model the circulation and interaction of influenza virus strains combining population ecology with immuno-epidemiology. Wu et al. [39] linked microbial ecology to systems biology by modeling quorum-sensing replication pathways through dynamic networks.

Contemporary trends in scientific research have unified such models using the “One Health” concept. Mathematical frameworks for multi-host pathogen dynamics and networks for disease spread were initially developed by Ferguson et al. [43] and Keeling and Rohani [44]. Current frameworks are becoming more and more focused on integrating environmental factors, animal vector host pools, and human clinical data for building an effective disease surveillance pipeline. Such developments illustrate how mathematical ecology contributes to international goals of sustainable development through Good Health and Well-being (SDG 3), Clean Water and Sanitation (SDG 6), and Life on Land (SDG 15).

#### Theoretical and mathematical ecology

The Theoretical and Mathematical Ecology category consists of the fundamental analytical tools used in the field. Examples of such works include Codling et al. [37], Liu et al. [41], and Lyons et al. [13]. Codling et al. [37] introduced the idea of the random walk theory as a mathematical paradigm for animal movement and spatial ecology. Liu et al. [41] developed generalized mathematical paradigms for network resilience based on ideas from statistical physics, applied to multi-trophic ecological networks. Lyons et al. [13] generalized MacArthur–Wilson island biogeography to microscopic level and provided simulations to support microbial biodiversity dynamics in suspended organic material.

Modern research builds further on such theoretical framework. In their research, Benitez-Lopez et al. [14] integrate the allometric scaling theory together with the evolution dynamics for testing the validity of the island rule in vertebrates. The spatial metapopulation theorems by Hanski [30], which modern researchers use as a basis for finite-size scaling to uncover ecological tipping points and regime shifts, also contribute to sustainability from a theoretical point of view. They contribute to SDG 7 (Affordable and Clean Energy), SDG 9 (Industry, Innovation and Infrastructure), and SDG 12 (Responsible Consumption and Production).

While earlier fundamental advances were primarily built on ideal deterministic differential systems and unconstrained random walks (Codling et al. [37]), recent advances in the methodological approach (2020–2025) illustrate a significant tendency towards stochastic individual-based models, physics-aware machine learning, and complex networked metapopulations. Modern approaches generalize classic stability approaches (e.g., Rosenzweig and MacArthur [6]) and the metapopulation approach to space [30], incorporating heterogeneous spectra of networks and finite-size scaling. In this way, modern models can unify nonlinear bifurcations and stochasticity of the environment with empirical time series.

#### Thematic map analysis

The thematic map of science is a two-dimensional strategic depiction of research themes through plotting the **degree of relevance (centrality)** on the x-axis and the **degree of development (density)** on the y-axis [19]. Centrality is an index that evaluates the degree of interactions and cohesion of a given cluster with other elements of the network, denoting the importance of the theme structurally in the discipline. The density is the measure of the internal intensity and cohesion of connections inside a cluster, denoting the maturity of the theme and its developmental trajectory. These form four quadrants:

1. **Motor Themes (Top-Right):** Very advanced and structurally important groups that generate intellectual dynamics for the field.
2. **Basic Themes (Bottom-Right):** Fundamental and transcending the discipline themes that have high structural centrality and low density.
3. **Niche Themes (Top-Left):** Very specialized and internally developed fields which are highly localized but poorly externally centralized.
4. **Emerging/Declining Themes (Bottom-Left):** Groups having low density and low centrality, indicating new trends or fading areas.

**Fig. 4:**
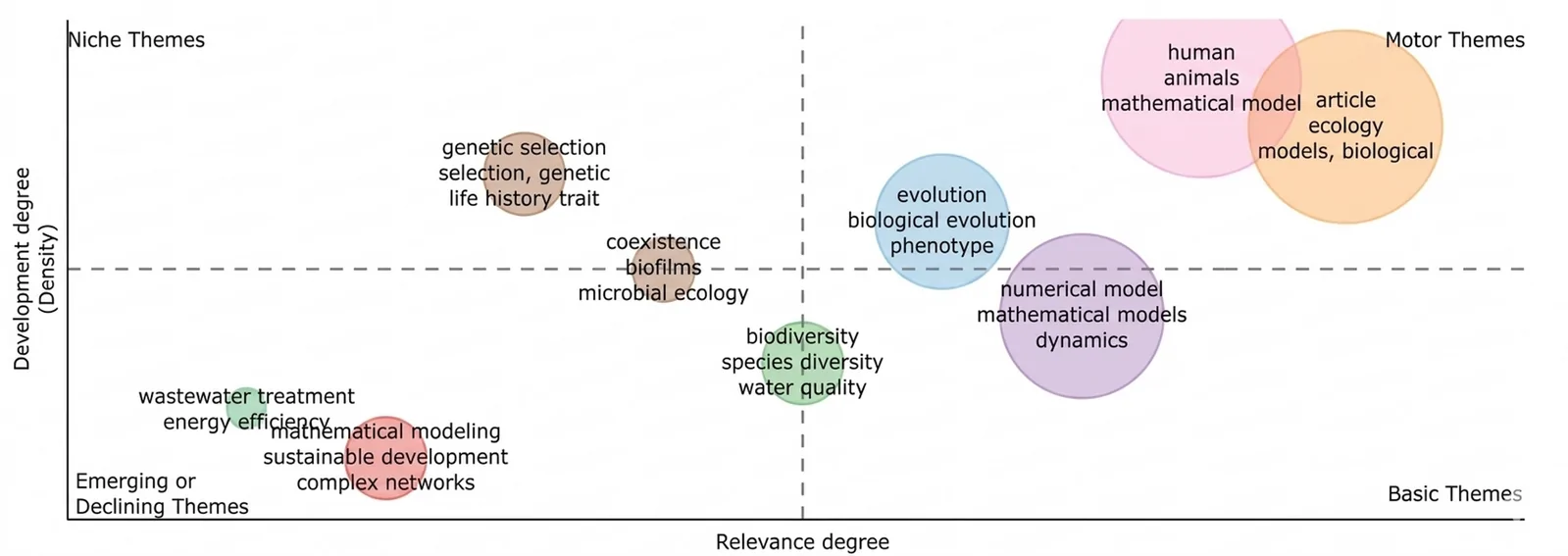
Thematic map of research themes in ecological modeling (bibliometrix R-package)

The thematic map created from the empirical theme analysis in the corpus of 2005–2025 is represented in Fig. 4. The motor themes situated in the upper right-hand corner are based on the most influential cluster of *humans, animals, mathematical model, article, ecology, and models, biological*. The positioning of these themes emphasizes the high level of integration of the ecological modeling with epidemiology and public health worldwide. Classical methodology frameworks including classical compartmental epidemic models (SIR/SEIR) [45], basic reproduction number models [46], and current spatial simulation models [43, 44] are used as the engines of practice.

The basic concepts, represented in the lower-right quadrant, form the fundamental methodology of the field. A group of topics including *numerical model, mathematical models,* and *dynamics* is found in this quadrant. Such concepts serve as the essential mathematical framework of ecology based on deterministic predator-prey models [6], stability criteria [47], and spatial metapopulations [48]. Although important across various subdisciplines of ecology, the placement of these concepts results from their methodological evolution through numerical methods and stochastic differential models [8].

Niche themes (upper-left quadrant) are highly specialized but relatively coherent and located at the periphery of the entire field. Groups of *genetic selection, life history traits, microbial ecology, biofilms,* and *coexistence* belong to this quadrant. The themes in question are empirical and lab-based due to classical competitive exclusion experiments [49], mathematical models of genetic selection [7], allometric body-size scaling laws [14], and plant resource-competition dynamics [10]. Although highly rigorous, their specialized nature keeps them structurally distinct from broad-scale macroecological modeling.

Themes in the bottom left corner are distinguished by their low centrality and density, which indicates that they may be either emerging frontiers or fading concepts. The clusters that contain themes like *wastewater treatment, energy efficiency, sustainable development,* and *complex networks* are found in this region. Despite the fact that these subjects were peripheral in classical naturalistic ecology, they constitute emerging interfaces.

In order to clarify whether the clusters positioned in the lower left quadrant correspond to emerging or declining themes, a temporal slice analysis is performed by comparing the earlier (2005–2014) and current decades (2015–2024):

- **Emerging Themes:** Clusters relating to complex network systems, sustainable development, and eco-hydrological modeling witnessed a * > 240%* rise in keyword frequency relative to 2015–2024 and thus showed a strong sense of future direction based on the computational graph theory and systemic sustainability requirements.
- **Declining Themes:** On the other hand, separate keywords like wastewater treatment and energy efficiency dropped in relative frequency since they have been transferred to specialized chemical engineering and environmental technology publications.

Lastly, intermediate clusters including *evolution, biological evolution, phenotype, biodiversity, species diversity,* and *water quality* are positioned around the central axis. Such even placement indicates that these clusters function as mediators between biological and computational simulations, allowing for mathematical expressions to be realistic from the point of view of physiology and ecology. Overall, the thematic map provides an illustration of how mathematical ecology connects motor epidemiological frameworks, numerical dynamics, biological niche data, and systemic sustainability architecture.

### SDG mapping and interlinkages

Research Question 3 assesses the alignment of peer-reviewed literature in mathematical ecology in light of the United Nations Sustainable Development Goals (SDGs). Scopus and Elsevier SciVal were used to address the research question. The UN Sustainable Development Goals include 17 main goals and 169 measurable targets. Previous bibliometric studies showed the efficiency of assessment of the scientific corpus in the light of these sustainability criteria [22, 23]. In this research, publications were classified using the Elsevier SDG Classification Tool [24]. Elsevier SDG Classification Tool is included in the Scopus as Science-Metrix. This tool consists of 17 search strings which were prepared especially for each of the goals according to their criteria. Machine Learning algorithm pre-trained on the corpus was used for better precision of classification, and its accuracy is higher than 80% [50].

**Fig. 5:**
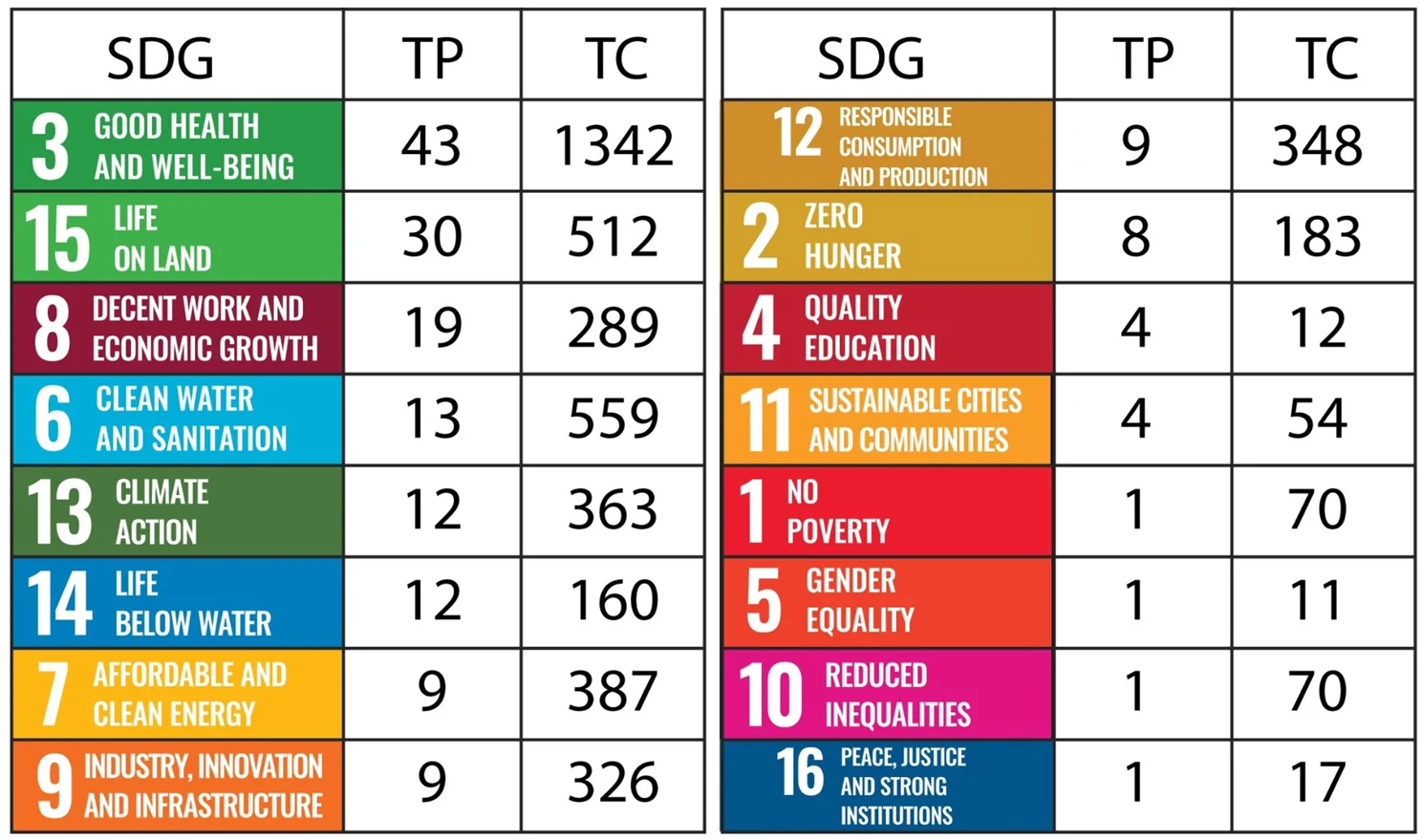
Distribution of publications across SDGs (SciVal)

Based on the Elsevier SDG mapping pipeline, 116 articles were found to align directly with one or more SDGs. The goals exhibiting the highest representation include Good Health and Well-being (SDG 3, *n=43*), Life on Land (SDG 15, *n=30*), and Decent Work and Economic Growth (SDG 8, *n=19*). Figure 5 presents the complete distribution of mapped publications and their cumulative citation accrual. In terms of citation impact, SDG 3 leads with 1,342 citations, followed by Clean Water and Sanitation (SDG 6, 559 citations) and Life on Land (SDG 15, 512 citations).

To evaluate the domain-specific classification accuracy of the pre-trained Elsevier-SciVal SDG machine learning model on our corpus, a stratified random audit of 20 mapped publications (*∼*17.2% of the 116 SDG documents) was conducted by two independent annotators. Each paper was manually evaluated against the official United Nations 2030 Agenda targets and indicators. The audit demonstrated a True Positive Rate (Sensitivity) of 85.0% (*n=17/20*) and a False Positive Rate of 10.0% (*n=2/20*), with one borderline classification. This confirms that the automated classifier operates with robust fidelity in domain-specific mathematical ecology literature.

While climate change is widely acknowledged as an existential ecological driver, publications mapping directly to SDG 13 (Climate Action) ranked fifth in volume (*n=12*, 363 citations), trailing Good Health and Well-being (SDG 3, *n=43*) and Life on Land (SDG 15, *n=30*). This relatively low presence in our sample does not mean that ecological models do not account for climatic factors; rather, it shows that climatic factors (such as changes in temperatures and thermal tolerance) are mostly modelled as second-order exogenous factors for the context of terrestrial biodiversity (SDG 15) or vector-borne disease ecology (SDG 3), not as an independent SDG 13 intervention.

#### SDG interlinkages

An analysis of the linkages between SDGs is very important for systemic environmental management because, by their nature, the UN goals are all interconnected and not disjointed [51]. The mapping of multiple goal linkages shows how different sustainability areas are affected at once by quantitative models.

**Fig. 6:**
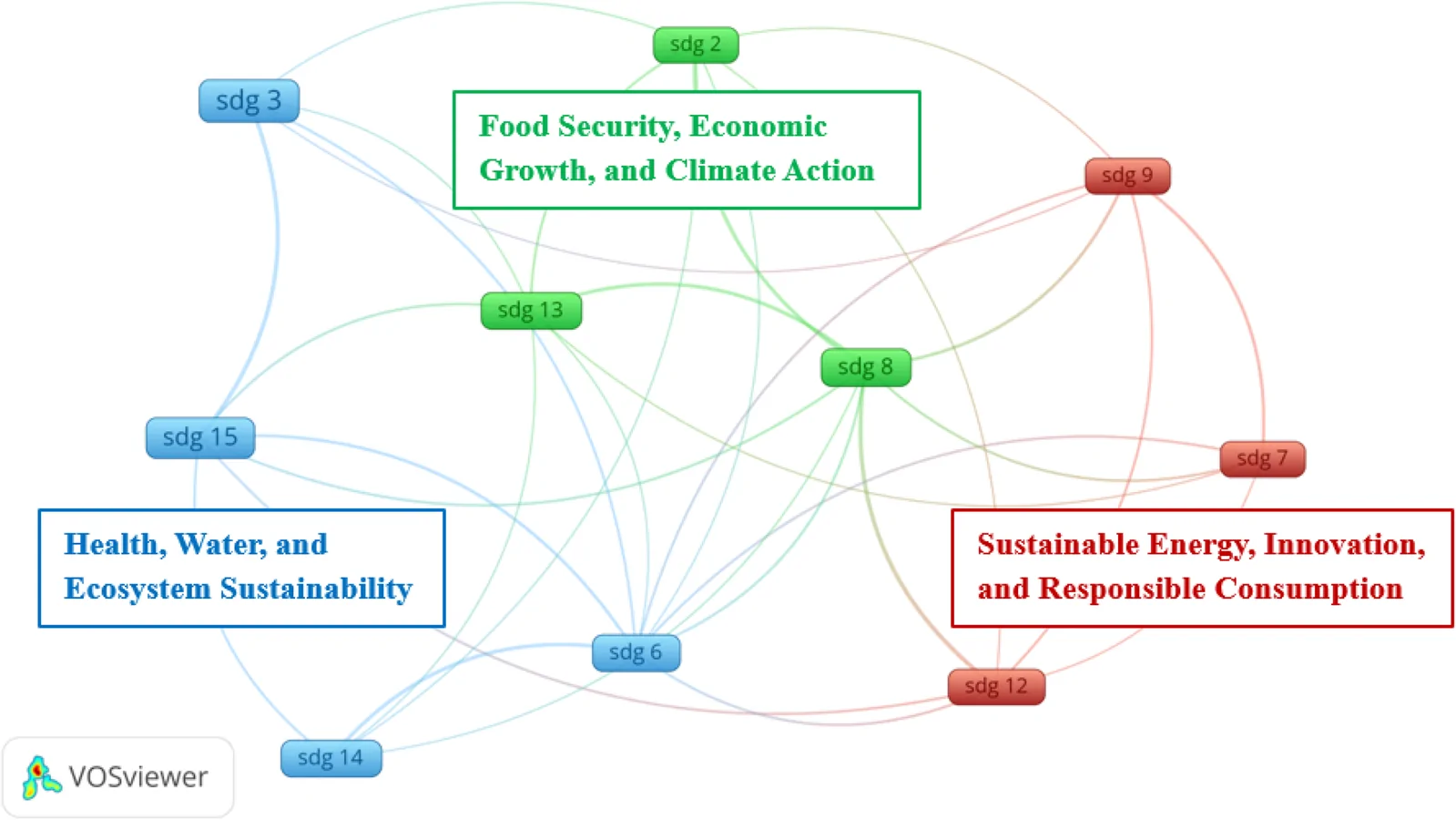
SDG interlinkage network generated via VOSviewer

Figure 6 visualizes the empirical co-occurrence network of mapped SDGs across the analyzed corpus. Each node represents an individual Sustainable Development Goal, and the connecting edges delineate thematic interconnections and multi-target modeling applications.

In the SDG interlinkage network (Fig. 6), nodes represent individual Sustainable Development Goals, scaled proportionally to publication volume. Edges between nodes represent empirical co-occurrence within identical document metadata—i.e., when an individual study satisfies the Elsevier-SciVal search string for multiple SDGs simultaneously. Edge weights correspond directly to the frequency of shared documents ($w_{ij} = \sum D_{i \cap j}$). Of the 116 SDG-aligned articles, 34 demonstrated multi-goal co-occurrences, with the strongest edge weight occurring between SDG 3 and SDG 15 (*w=11*), followed by SDG 6 and SDG 14 (*w=7*), reflecting the integrated nature of One Health and aquatic-terrestrial water governance.

Projecting the three core thematic clusters from section “Thematic modeling” onto the SDG network highlights clear translational alignments. The *Biological Foundations* cluster (Green) corresponds to **Food Security, Economic Growth, and Climate Action** (SDGs 2, 8, 13); the *Epidemiological and Human Dimensions* cluster (Blue) connects to **Health, Water, and Ecosystem Sustainability** (SDGs 3, 6, 14, 15); and the *Mathematical and Computational Approaches* cluster (Red) aligns with **Sustainable Energy, Innovation, and Responsible Consumption** (SDGs 7, 9, 12). These bipartite interconnections are illustrated in Fig. 6.

#### Sustainable energy, innovation, and responsible consumption (red cluster)

Mathematical and computational formulations map directly to SDGs 7, 9, and 12, where quantitative algorithms drive advancements in technology, infrastructure resilience, and resource efficiency. Energy-flow formulations in environmental engineering inform low-carbon transitions and bioenergy systems, supporting SDG 7 by optimizing energy generation and thermal dynamics [52, 53]. Advanced computational techniques—including network science, agent-based architectures, and dynamical systems modeling—provide methodological infrastructure that advances SDG 9 by supporting sustainable engineering design [54]. Concurrently, dynamic carrying capacity models, material flow analyses, and pollutant dissipation equations provide empirical guidance for circular industrial workflows and waste minimization under SDG 12 [55]. These findings illustrate that computational ecology provides practical algorithms for industrial circularity and green technology development.

#### Food security, economic growth, and climate action (green cluster)

The biological foundations and empirical data of ecological modeling map onto SDGs 2, 8, and 13, establishing the scientific baseline for agrarian stability, nature-based economies, and climatic resilience. Multi-trophic food-web models and resource competition equations inform agroecosystem management, optimizing crop yields, suppressing pest outbreaks, and preserving soil health to support SDG 2 under shifting climatic baselines [56]. Landscape heterogeneity simulations, dynamic biocapacity accounting, and ecosystem service valuation models underpin SDG 8 by evaluating sustainable rural livelihoods where local economies depend on ecological capital [57]. Furthermore, climate-coupled ecological formulations evaluate non-linear ecosystem thresholds, thermal tolerances, and adaptive capacities, supplying predictive evidence required for mitigation and adaptation frameworks under SDG 13 [58]. Collectively, empirical biological parameterization is vital for safeguarding food security and guiding climate-compatible development.

#### Health, water, and ecosystem sustainability (blue cluster)

Cluster of epidemiology and human dimensions is relevant to SDGs 3, 6, 14, and 15 that identify the importance of using ecological models for the protection of humans, aquatic life, and terrestrial biodiversity. Models for the compartmental spread of disease, the surveillance system design of zoonoses, and transmission networks contribute to SDG 3 in terms of providing a warning mechanism and assessment of vector control measures [59]. Hydro-ecological transport models and simulations of biomass in the aquatic ecosystem help SDGs 6 and 14 with assessing the capacity of water purification, discharge of groundwater, and food webs in marine ecosystems from anthropogenic pollution [60, 61]. On the other hand, population viability analysis, disturbance–biodiversity relations, and habitat fragmentation modeling are relevant to SDG 15 in assessing species’ risk of extinction and regime shifts [62].

#### Interlinkages across clusters

These cross-cluster linkages illustrate the fact that ecological modeling is a systems science in which individual actions affect multiple targets at once. For example, Climate Action (SDG 13) serves as a major biophysical driver for terrestrial biodiversity (SDG 15), agriculture production (SDG 2), and the transition to clean energy sources (SDG 7). Equally, Clean Water and Sanitation (SDG 6) links the aquatic life (SDG 14) with public health epidemiology (SDG 3) and terrestrial watershed management (SDG 15). These multi-objective interactions show that local mathematical models—from microbial reactions to multispecies predator-prey relations—serve as the numerical foundations necessary to design global sustainability strategies.

## Implications for ecological mathematical modeling

The growing use of mathematical models in ecology brings with it an inherent operational problem; although accurate predictive models are essential for formulating effective sustainability policies, data limitations, structural assumptions, and applicability of specific models prevent their practical use. With the increasing strain caused by loss of biodiversity, depletion of natural resources, and climate change-induced degradation of ecosystems, environmental policy makers should integrate integrated predictive modeling tools into national climate policies and sustainable development goal monitoring frameworks in order to achieve resilient food production (SDG 2), climate-compatible economic growth (SDG 8 and 13), and terrestrial and aquatic conservation (SDG 14 and 15) [56, 58, 62]. Mathematical ecological models and predictive early-warning indicators serve as the mechanistic basis needed to move from reactive environmental management to proactive environmental management. This can be done by modeling the trade-offs that arise from anthropogenic resource usage and the delivery of ecosystem services. In this sense, integrated hydro-ecological models and water quality models serve as useful tools in governing watersheds sustainably as part of SDG 6 [60] and dynamic carrying capacity models and complex networks can help with governing infrastructure development and circular industry production within the framework of SDGs 9 and 12 [55]. It is essential to have robust protocols of model validation, including uncertainty quantification and global sensitivity analysis.

As opposed to the assumption that these policies will simply be taken up by the multilateral treaty secretariats directly and without intermediation, such policies have to be fostered through national scientific research granting councils and regulatory requirements and academic incentives. These organizations need to set aside interdisciplinary grants in order to bring together theoretical biomathematicians and computational ecologists with local environmental protection agencies, basin boards, and public health institutions. Prioritizing such methodologies within grant calls in terms of delivering early warning systems for disease vectors or seasonal catchment runoff modeling guarantees practical outcomes.

Equally important is achieving global equity and geographic inclusiveness in ecological modeling. Emerging economies, especially within the Global South, tend to have serious computational constraints as well as critical data gaps from the field, potentially resulting in an unequal and disjointed journey towards realizing the goals set out in the 2030 Agenda. Special efforts, such as establishing open-source ecological data repositories, creating regional computing consortia, and organizing south-to-south modeling workshops, are needed in order to address such inequalities. International organizations like IPBES and UNEP need to create a shared infrastructure for computational purposes in order to conduct uniform and climate resilient ecological modeling as well as SDG reporting among resource-limited countries. Finally, turning mathematics into an actual tool in ecology will require bridging the communication gap that exists between the theorists, the practitioners, and the policymakers.

### Operationalizing ecological modeling: a multi-tiered framework for SDG intervention

In order to address the empirical deficiencies present in various underrepresented and undersampled ecosystems and to convert theoretical concepts into concrete policies, we recommend a scalable three-step approach, which is as follows:

- **Tier 1 (Data Collection & Harmonization):** Continuous ingestion and harmonization of open-source earth-observation datasets (e.g., Copernicus, Landsat, Sentinel) alongside community-science biodiversity datasets (e.g., GBIF, iNaturalist) in order to cover for sparse terrestrial observation network infrastructure.
- **Tier 2 (Hybrid Mechanistic-Stochastic Model Engine):** Modelling framework comprising modular integration of mechanistic deterministic differential equations (e.g., multi-host compartmental epidemic models for SDG 3, trophic food web models for SDG 15) along with physics-informed neural networks (PINN) and stochastic jumps in order to guarantee the fidelity of the model and its predictability despite the severe lack of data.
- **Tier 3 (Policy-Interface Dashboard):** High-dimensional model output conversion into user-friendly sustainability metrics (e.g., predicting localized spillover of pathogens based on planned deforestation activities for Target 3.3, as well as carrying capacity under severe drought conditions for Target 6.4).

**Translational Pathways from Model to Policy:**

1. **For SDGs 3 & 15 (One Health Vector Integration):** We recommend that municipal and national public health agencies formally embed compartmental eco-epidemiological models (Cluster 2) into operational disease surveillance pipelines. By coupling time-varying vector reproduction numbers ($R_0$) with satellite-derived land-use disturbance and canopy-loss metrics, public health authorities can delineate real-time early-warning zones for zoonotic spillover along rapidly expanding agricultural–forest ecotones.
2. **For SDGs 6 & 14 (Catchment and Aquatic Governance):** Regional river basin authorities should mandate coupled hydrodynamic–biogeochemical ecological models (Cluster 3) as prerequisite components for industrial and agricultural discharge permits. This enables authorities to establish dynamic, seasonal pollutant load thresholds based on aquatic food-web resilience rather than relying on static chemical concentration limits.

**Synthesis of Resolved Research Gaps:** The empirical findings and frameworks presented in this study directly resolve the four foundational research gaps established in Table 1:

1. **Lack of structured thematic synthesis:** Resolved via the modular three-cluster keyword co-occurrence topology (section “Thematic modeling”), which delineates the biological foundations, epidemiological dimensions, and theoretical mathematical dynamics structuring the discipline.
2. **Absence of bibliometric science mapping in mathematical ecology:** Resolved through the two-dimensional density–centrality thematic map (Fig. 4), characterizing motor, basic, niche, and emerging/declining trajectories over two decades.
3. **Absence of SDG mapping:** Resolved by empirically classifying 116 SDG-aligned publications and revealing the dominant concentration in Good Health and Well-being (SDG 3), Life on Land (SDG 15), and Decent Work and Economic Growth (SDG 8) (Fig. 5).
4. **Integration of thematic structure with societal sustainability priorities:** Resolved via the multi-cluster bipartite interlinkage network (Fig. 6), which connects quantitative modeling pillars directly to actionable UN 2030 targets.

**Methodological Boundaries and Database Dependency:** An important boundary condition for the current study involves the practical use of the Scopus bibliographic database. Despite the comprehensiveness of the coverage of Scopus in terms of applied mathematics, computational biology, and environmental sciences, in addition to having the built-in capacity for validating the SciVal UN SDG classification algorithm, the database has differences in its coverage compared to Web of Science Core Collection, PubMed, and IEEE Xplore. This implies that some specialized mathematical theory publications or biomedical publications may not be included if they are indexed only in other bibliographic databases. Future science-mapping investigations combining multiple bibliographic databases through automated deduplication pipelines are encouraged to verify cross-database structural consistency.

## Conclusion

In the present study, we perform a systematic twenty-year bibliometric and thematic analysis of peer-reviewed research conducted at the interface between mathematical modeling and ecology. By analyzing 229 Scopus-indexed papers published during the period from 2005 to 2025, we trace the dynamic intellectual structure between biological experimental data, mathematical approaches, and epidemiological monitoring. Statistical indicators of productivity illustrate that mathematical ecology is a well-established discipline with a stable methodology marked by constant scientific activity, effective collaboration between authors worldwide, and significant citation impact. Thematic mapping highlights that the motor themes of mathematical ecology rely strongly on public health epidemiology and compartmental disease modeling, while basic themes form the necessary theoretical and mathematical base. On the contrary, sustainable and eco-hydrological network modeling are promising areas for development.

The empirical mapping of SDGs reveals strong alignment with public health (SDG 3, *n=43*), terrestrial conservation (SDG 15, *n=30*), and nature-based economic provisioning (SDG 8, *n=19*). Contrary to previous views that there was an inherent deficit in research related to all technological SDGs, computational modeling is already being applied in bioenergy and resource dynamics (SDGs 7 and 9, *n=9* each). However, there remain important themes overlooked at the interface of technology and ecology. First, mathematical modeling is poorly applied in the evaluation of renewable energy land-use impacts, industrial ecology circularities, limitations of smart grids, and life cycle ecological impacts. To have a societal impact, mathematical ecology needs to evolve from a purely descriptive science to decision support through predictive models in national climate change adaptation strategies, municipal One Health monitoring systems, and watershed permits.

**Limitations and Future Research Directions:** The findings and conclusions of this study should be interpreted within the context of four inherent methodological boundary conditions:

1. **Search Syntax Specificity:** Literature searching was limited to the publications containing exact phrase pair co-occurrence of “Mathematical Modeling” and “Ecology” in either title or abstract. Although this particular search syntax ensures high topical consistency and is confined to only the core self-identified literature, it also excludes the literature using entirely different specialized terminology (e.g., “computational biology” or theoretical population ecology papers not featuring the “ecology” keyword).
2. **Single-Database Reliance:** The bibliometric data collection was conducted using Elsevier Scopus to retain integration with proven SciVal UN SDG classification algorithms. Therefore, high-impact articles published in the Web of Science Core Collection, PubMed, or other specialized mathematical databases were not included in the study.
3. **Document-Type Delimitation:** Analysis was confined to peer-reviewed journal articles and reviews to retain metadata uniformity and citation comparability while excluding conferences, monographs, and edited books potentially containing new algorithms.
4. **Temporal Cut-Off Boundary:** The inclusion window covered literature up to August 6, 2025. Although using a partial-year time period ensured the coverage of the most immediate cutting-edge research, the publication volume of 2025 was not due to an academic productivity downturn but partial-year coverage.

Future research should deploy multi-database automated deduplication pipelines combining Web of Science, Scopus, and Dimensions, while integrating natural language processing (NLP) and dynamic topic modeling (e.g., BERTopic) to capture latent linguistic shifts across broader, unconstrained computational ecology corpora.

## Data Availability

The complete bibliographic dataset of 229 analyzed articles—including digital object identifiers (DOIs) is publicly accessible via the Zenodo data repository (DOI: https://doi.org/10.5281/zenodo.22733116).
